# Rosiglitazone Mitigates Dexamethasone-Induced Depression in Mice via Modulating Brain Glucose Metabolism and AMPK/mTOR Signaling Pathway

**DOI:** 10.3390/biomedicines11030860

**Published:** 2023-03-11

**Authors:** Aisha Alhaddad, Asmaa Radwan, Noha A. Mohamed, Eman T. Mehanna, Yasser M. Mostafa, Norhan M. El-Sayed, Shaimaa A. Fattah

**Affiliations:** 1 Department of Pharmacology and Toxicology, College of Pharmacy, Taibah University, Al-Madinah Al-Munawwarah 30078, Saudi Arabia; 2 Department of Pharmacology &Toxicology, Faculty of Pharmacy, Suez Canal University, Ismailia 41522, Egypt; 3 Department of Forgery & Counterfeiting, Forensic Medicine, Ministry of Justice, Ismailia 41522, Egypt; 4 Department of Biochemistry & Molecular Biology, Faculty of Pharmacy, Suez Canal University, Ismailia 41522, Egypt; 5 Department of Pharmacology & Toxicology, Faculty of Pharmacy, Badr University in Cairo, Badr 11829, Egypt

**Keywords:** rosiglitazone, major depressive disorder, dexamethasone, glucose metabolism, AMP-activated protein kinase, mitogen-activated protein kinases, mTOR protein, hexokinase, pyruvate kinase, NGF

## Abstract

Major depressive disorder (MDD) is a common, complex disease with poorly understood pathogenesis. Disruption of glucose metabolism is implicated in the pathogenesis of depression. AMP-activated protein kinase (AMPK) has been shown to regulate the activity of several kinases, including pAKT, p38MAPK, and mTOR, which are important signaling pathways in the treatment of depression. This study tested the hypothesis that rosiglitazone (RGZ) has an antidepressant impact on dexamethasone (DEXA)-induced depression by analyzing the function of the pAKT/p38MAPK/mTOR pathway and NGF through regulation of AMPK. MDD-like pathology was induced by subcutaneous administration of DEXA (20 mg/kg) for 21 days in all groups except in the normal control group, which received saline. To investigate the possible mechanism of RGZ, the protein expression of pAMPK, pAKT, p38MAPK, and 4EBP1 as well as the levels of hexokinase, pyruvate kinase, and NGF were assessed in prefrontal cortex and hippocampal samples. The activities of pAMPK and NGF increased after treatment with RGZ. The administration of RGZ also decreased the activity of mTOR as well as downregulating the downstream signaling pathways pAKT, p38MAPK, and 4EBP1. Here, we show that RGZ exerts a potent inhibitory effect on the pAKT/p38MAPK/mTOR/4EBP1 pathway and causes activation of NGF in brain cells. This study has provided sufficient evidence of the potential for RGZ to ameliorate DEXA-induced depression. A new insight has been introduced into the critical role of NGF activation in brain cells in depression. These results suggest that RGZ is a promising antidepressant for the treatment of MDD.

## 1. Introduction

In recent decades, millions of individuals around the globe have been adversely affected by depression, making it one of the most prevalent neuropsychiatric illnesses [1]. The underlying mechanism of depression is still obscure. Patients with depression typically display depressive behavior together with certain pathological signs, including aberrant cytokine release, inflammation, and deficits in cell proliferation and neuroplasticity [2]. Because the central nervous system is an immunologically privileged area, it can produce inflammatory substances that help keep the immune system intact. The development of long-term immunological tolerance leads to a markedly increased secretion of inflammatory components, which are associated with inflammatory exacerbation and, consequently, with the pathogenesis of a wide spectrum of neurological and psychiatric disorders. There is ample evidence that inflammatory cytokines are significantly elevated in the serum of MDD patients [3]. Unpredictable chronic mild stress may cause depressive behaviors in rats, so it is frequently used to model depression in animals [4]. Chronic stress triggers the secretion of corticosterone and glucocorticoids in both humans and animals, and depression occurs with the release of high concentrations of corticosterone [5].

Two important brain regions regarding depression are the prefrontal cortex and the hippocampus. The hippocampus is critical to the brain’s learning and memory processes [6,7]. Previous research has shown that changes in the hippocampal morphology during depression impair its function and memory retrieval [8,9]. The prefrontal cortex has been shown to play an important role in social-emotional processing [10]. Frontal alpha asymmetry showed that major depression disorder (MDD) patients appeared to have increased alpha power in the left frontal hemisphere and decreased alpha power in the right hemisphere compared with control subjects [11]. When a person becomes depressed, he or she leaves the typical waking state and enters a separate global level of consciousness, such as dreaming or having psychedelic experiences [12]. It was suggested that qEEG and functional connectivity methods can be used to predict and characterize patients with disorders of consciousness in terms of their rehabilitative capacity and functional recovery [13].

It is believed that anomalies in brain glucose metabolism have a role in the etiology of depression. Most of the brain’s energy comes from glucose. The optimum neurotransmitter synthesis and function, in particular the production of glutamate and -aminobutyric acid (GABA), as well as the production of enough NADPH, are made possible by glucose metabolism [14]. The frontal brain and hippocampus of depressed mice showed greater glucose/glycogen, and glucose transporter (GLUT1–GLUT3) levels than healthy animals due to the intensity of glucose uptake during depression [15]. A previous study examined the expression of glycolysis and glycogen-related genes in the hippocampus of depressed rats, and the results showed that the mRNA level of Slc2a3 (encoding GLUT3) increased [16]. According to certain hypotheses, the risk of developing mental, metabolic, and cardiovascular disorders is elevated by prolonged stimulation of the hypothalamic-pituitary-adrenal (HPA) axis. In fact, accumulating clinical evidence consistently demonstrates a link between negative cardiovascular events, obesity, type 2 diabetes mellitus (DM), metabolic syndrome, and hypertension [17,18]. Available evidence shows that the principal cause for the coexistence of MDD due to metabolic disturbances is the excessive action of glucocorticoids [19]. Given that there is a significant correlation between depression and metabolic disorders, particularly DM, several studies have investigated the anti-depressive effects of some antidiabetic medications as a trial to develop new therapeutic approaches for the management of depression [20]. Diabetes seems to be a risk factor for the incidence of depression, and a quarter of diabetic subjects show depressive symptoms [21]. Numerous antidiabetic medications, including metformin, thiazolidinedione, and sulfonylureas, have either been shown or are supposed to alleviate depression in such circumstances [22,23,24].

Rosiglitazone (RGZ), a member of the thiazolidinedione group that is frequently prescribed as an insulin-sensitizing drug to alleviate type 2 DM, has been shown to exhibit neuroprotective action in numerous central nervous system disorders [25], including Parkinson’s disease [26], Alzheimer’s disease [27], and stroke [28]. Intriguingly, pioglitazone, which has a similar chemical composition to that of RGZ, has been shown to alleviate depression [29]. Moreover, Patel et al. [30] reported that RGZ effectively helped in the management of neurological diseases accompanied by depression-like behavior. However, it is still unclear what processes underlie these effects.

In humans, physiological brain glucose increases in parallel with peripheral hyperglycemia [31]. High glucose, shown to be an independent risk factor for insulin resistance in human neuroblastoma cells, rat primary cortical neurons, and the cerebral cortex of hyperglycemic db/db mice, promotes mitochondrial dysfunction, adenosine monophosphate-activated protein kinase (AMPK) inactivation, and mTOR/AKT activation [32].

Mammalian target of rapamycin (mTORC) is a serine/threonine kinase that is expressed during dendritic development, where mTOR regulates the initiation of new protein translation and subsequent translation [33]. Dysfunction of mTOR is involved in the etiology of different neuropsychiatric disorders, including depression [34]. Recently, it was revealed that the antidepressant effect mediated by the mTOR/AMPK pathway is accomplished by increasing autophagy in chronic unpredictable mild stress mice [35,36]. These findings suggest a possible novel approach for the treatment of depression related to the AMP/ATP ratio and AMPK. Therefore, this study investigated the effect of RGZ on AMPK, mTOR, and their downstream signaling factors adenosine monophosphate-activated protein kinase (pAKT), mitogen-activated protein kinases (p38MAPK), and eukaryotic initiation factor 4E (eIF4E)-binding proteins (4EBP1) in a depressed mouse model.

Olson described the growth factor known as nerve growth factor (NGF) as a neurite outgrowth factor [37]. Studies proved that NGF is implicated in neuronal repair and survival [38,39]. The role of NGF in MDD is still understudied. In the Flinders Sensitive Line (FSL) rat model of depression, electroconvulsive therapy (ECT) increased NGF levels in the hippocampus [40]. Furthermore, subcutaneous NGF injections have been reported to alleviate depression [41].

In the current work, the mechanisms underlying the antidepressant effect of RGZ, such as regulation of AMPK and mTOR, and their downstream signaling pathways of pAKT, p38MAPK, 4EBP1, and NGF, were investigated in a mouse model of depression induced by dexamethasone (DEXA).

## 2. Materials and Methods

In this study, the antidepressant effect of RGZ was investigated in mice that received a subcutaneous dose of DEXA (20 mg/kg) for 21 days to induce MDD-like pathology. The expression of GLUT 1, GLUT 3, pAMPK, pAKT, p38MAPK, and 4EBP1, and the levels of hexokinase, pyruvate kinase, and NGF were examined in the prefrontal cortex and hippocampus tissue samples to explore the potential mechanism of RGZ.

### 2.1. Animals

In the current study, 32 Swiss Albino male mice weighing 25–30 g were used. The sample size was calculated by the resource equation method [42]. Mice were kept in a temperature-controlled environment (22 ± 3 °C) with a regular 12:12 h light:dark cycle, normal food, and unlimited access to water. Prior to the investigation, mice were given at least 5–7 days to acclimate. Throughout the experiment, mice were weighed and regularly monitored for any indications of distress. The experiment protocols were performed in compliance with the Animal Ethics Committee’s guidelines at the Faculty of Pharmacy, Suez Canal University (License number 201609RA1), in accordance with the Canadian Council on Animal Care Guidelines.

### 2.2. Drugs

RGZ was kindly provided by Medical Union Pharmaceuticals (Abou Sultan-Ismailia, Egypt) and was suspended in 1% sodium carboxymethyl cellulose (Na-CMC) to achieve a final concentration of 1%. DEXA was acquired from Sigma-Aldrich (St. Louis, MO, USA) and suspended in saline (0.9% NaCl) containing 0.1% Tween-80 and 0.1% dimethyl sulfoxide (DMSO).

### 2.3. Induction of Depression

To induce depression-like behavior, three experimental groups were injected subcutaneously (s.c.) with DEXA (20 mg/kg) once daily for 21 consecutive days in a volume of 5 mL/kg at casual times during the light phase, whereas the normal control group received only vehicle as previously mentioned [43]. After three weeks, the development of the depressed model was confirmed through exposing mice to the tail suspension test (TST) and forced swimming test (FST). Mice displaying a greater immobility time in FST as well as TST had been considered depressed.

### 2.4. Experimental Design

Mice were randomly allocated to 4 weight-matched groups of eight mice each: Group 1: negative control group received vehicle (saline (0.9% NaCl) containing 0.1% Tween-80 and 0.1% DMSO); Group 2: positive control untreated “depressed group “ mice received DEXA 20 mg/kg; Group 3 and 4: depressed mice received daily s.c. DEXA 20 mg/kg and per oral (p.o.) doses of RGZ (10 or 30 mg/kg) for 21 days [44,45,46].

Following daily dosage, the TST and the FST were performed at the end of the experiment. Ketamine and xylazine were intraperitoneally injected at doses of 87.5 mg/kg and 12.5 mg/kg, respectively, to euthanize the mice via cervical dislocation. Each mouse’s frontal cortex and hippocampus were dissected, snap-frozen, and kept at −80 °C for subsequent analyses.

### 2.5. Forced Swimming Test and Tail Suspension Test

FST and TST are common methods for evaluating depression-like behavior [47]. The FST and TST were carried out in all experimental groups.

The FST was performed as previously mentioned [48,49]. Individually placed in 2000 mL glass beakers with 10 cm of water at room temperature (25 ± 1 °C), each mouse was given 5 min to swim. “Duration of immobility” refers to the time interval when mice are non-energetic and show none of the escape-oriented behaviors like swimming, diving, jumping, rearing, or sniffing. The immobility period was recorded during the last 4 min.

The TST was conducted as previously detailed [50]. Mice were allowed to rest for 24 h following FST. Mice were suspended from the edge of a shelf that was 58 cm above a tabletop by adhesive tape that was positioned about 1 cm from the tip of the tail. They were permitted to hang for a total of 6 min, and the duration of immobility was recorded during the test’s last 4 min. The only immobile mice were those that hung passively and motionlessly.

### 2.6. Enzyme-Linked Immunosorbent Assay

The levels of hexokinase and pyruvate kinase in the frontal cortex and hippocampus of mice were measured using Enzyme-Linked Immunosorbent Assay (ELISA) kits (EIAab Science Co., Ltd., Wuhan, China) following the manufacturer’s instructions. Hexokinase and pyruvate kinase concentrations were estimated and expressed as nmole/mg tissue.

NGF levels were assessed using a commercial ELISA kit specific for mice (Cusabio Technology, Houston, TX, USA). Absorbances were measured at 450 nm. NGF levels were expressed as pg/g tissue.

### 2.7. RNA Extraction and Real Time Quantitative PCR (RT-qPCR)

To measure the gene expression of GLUT1 and GLUT3 in the mice’s hippocampus and frontal cortex tissue, RNA was extracted using the SV total RNA isolation kit (Promega, Madison, WI, USA) according to the manufacturer’s instructions. Using a Nanodrop NA-1000 UV/vis spectrophotometer (Thermo Fisher Scientific Inc., Wilmington, DE, USA), RNA purity and concentration were measured and then stored at −80 °C. Messenger RNA (mRNA) transcript levels of GLUT1 and GLUT3 were quantified by real-time PCR using the StepOne Plus™ real-time PCR thermal cycler (Applied Biosystems, Waltham, MA, USA). RT-qPCR was performed using the GoTaq^®^ 1-Step RT-qPCR System (Promega, Madison, WI, USA) as previously described. The primers used are listed in Table 1. The thermal PCR amplification protocol was as follows: 37 °C for 15 min, 10 min at 95 °C, followed by 40 cycles of 95 °C for 10 s, 52 °C for 30 s, and 72 °C for 30 s. The generation of specific PCR products was confirmed through dissociation curve analysis. The threshold (Ct) values for each reaction were estimated. All the Ct values of the target genes were normalized to the Ct value of β-actin, which was used as a housekeeping gene.

### 2.8. Western Blotting Analysis

For the detection of pAKT (Ser473), pAMPK-α (Thr172), p38MAPK, 4EBP1, and mTOR, the frontal cortex and the hippocampus were homogenized in an ice-cold RIPA lysis solution supplemented with phosphatase and protease inhibitors to maintain the proteins integrity and phosphorylation. Lysates were centrifuged at 16,000× *g* for 10 min, and pellets were discarded. The supernatants were kept at −80 °C for subsequent analysis. The Bradford assay was performed to determine the concentrations of protein, as previously detailed [51]. The lysate was combined with an equal volume of 2 × Laemmli sample buffer, heated for 5 min at 95 °C, and then centrifuged for 10 min at 10,000× *g*. Resulting supernatants were subjected to 12% SDS–polyacrylamide gel electrophoresis. Proteins had been loaded to PVDF membranes using a Bio-Rad Trans-Blot Turbo system (Bio-Rad Laboratories Ltd., Watford, UK). Membranes were then blocked by incubation for 1 h in tris-buffered saline (TBS) containing 0.05% polyoxyethylenesorbitan monolaurate (Tween 20; TBS-T buffer) containing 5% (*wt*/*vol*) non-fat dry milk. Rabbit monoclonal anti-pAMPKα (Thr172) (Cat#ab 50081, cell signaling technology, 4228, Danvers, MA, USA), mouse monoclonal anti-pAKT (Ser473) (Cat#ab 4051, cell signaling technology, 4228, Danvers, MA, USA), rabbit monoclonal anti-p38MAPK (Cat#ab 8690, cell signaling technology, 4228, Danvers, MA, USA), rabbit monoclonal anti-4EBP1 (Cat#ab 9644, cell signaling technology, 4228, Danvers, MA, USA) or mouse monoclonal β-actin (Cat# ab3700, cell signaling technology, 4228, Danvers, MA, USA), were used as primary antibodies (1:1000 dilution in TBS-T with 5% non-fat milk) and membranes were incubated overnight at 4 °C. Blots were then subsequently washed in TBS-T and incubated with the HRP-conjugated secondary antibody; Goat Anti-Mouse IgG HRP-linked Antibody (Dako #P0447, Glostrup, Denmark), Goat anti-rabbit IgG HRP-linked antibody (1:5000 dilution, Cat#7074, Cell Signaling, 4228, Danvers, MA, USA). Membranes were incubated for 1 h at room temperature. Signals were visualized by chemiluminescence (Clarity^TM^ Western ECL substrate—BIO-RAD, Hercules, CA, USA cat#170- 5060) in accordance with the manufacturer’s instructions and recorded using a CCD camera-based imager. Densitometry was performed to measure the band intensities using the Image J program (version 1.48, National Institute of Health, Bethesda, MD, USA).

### 2.9. Statistical Analyses

In regard to the current study’s statistical analyses, the SPSS program’s version 24 was used (SPSS Inc., Chicago, IL, USA). Data were expressed as mean ± SD (standard deviation). A one-way analysis of variance (ANOVA) followed by Tukey’s post hoc multiple-comparisons test was applied, and a *p*-value of <0.05 was considered statistically significant.

## 3. Results

### 3.1. Improving Dexamethasone Induced Depression Behaviors

#### 3.1.1. Forced Swimming Test

**Figure 1:**
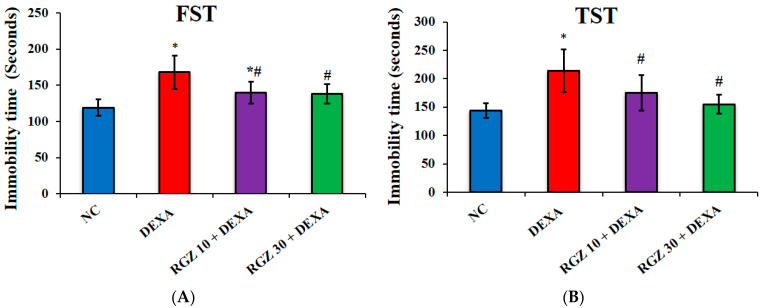
Effect of administration of DEXA, 20 mg/kg; s.c., RGZ, 10 mg/kg, or 30 mg/kg p.o. on the behavioral activity of FST (**A**) and TST (**B**) in mice. The statistical evaluation was conducted by a one-way ANOVA and then Tukey’s post hoc test. The values are represented by the mean ± SD (*n* = 8). NC—normal control; DEXA—dexamethasone; RGZ—rosiglitazone; FST—forced swimming test; TST—tail suspension test. A significant difference was considered at *p* < 0.05 and distinguished by * vs. NC; # vs. DEXA.

The one-way ANOVA analysis showed a significant difference in the mobility time recorded in the FST between the different groups (*F*_3,31_ = 15.2, *p* < 0.001). As shown in Figure 1A and Table S1, the untreated stressed mice displayed immobility behavior for 168 s, in contrast to 119 s in the normal mice (*p* < 0.001). When compared to the untreated group, mice that received DEXA in combination with 10 or 30 mg/kg RGZ showed a substantial decrease in immobility time of 16% (*p* = 0.003) and 18% *(p* = 0.001), respectively.

#### 3.1.2. Tail Suspension Test

A significant difference in the TST mobility time (*F*_3,31_ = 13.4, *p* < 0.001) was recorded. Post hoc analysis showed that the duration of immobility of the untreated DEXA group was significantly longer than that of the normal mice (214 and 144 s, respectively; *p* < 0.001). Following administration of both dosages of 10 and 30 mg/kg RGZ, immobility time was significantly reduced by 18% (*p* = 0.012) and 27.6% (*p* < 0.001), respectively, when compared with the DEXA group (Figure 1B and Table S1).

### 3.2. Reversal of GLUT1 and GLUT3 Expression Levels in Frontal Cortex and Hippocampus of Mice Post Dexamethasone Administration

Previous data indicated an association between depression and increased glucose metabolism in the hippocampus and frontal cortex [15,52]. In this context, the one-way ANOVA analysis indicated a significant difference in the expression of GLUT1 (*F*_3,31_ = 59.3, *p* < 0.001) and GLUT3 (*F*_3,31_ = 46.8, *p* < 0.001) in the different groups.

**Figure 2:**
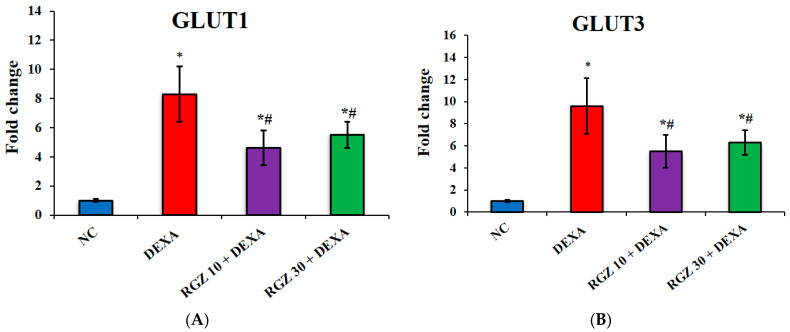
Effect of administration of DEXA, 20 mg/kg; s.c., RGZ, 10 mg/kg, or 30 mg/kg p.o., on GLUT1 (**A**) and GLUT3 (**B**) in the brain of mice. The statistical evaluation was conducted by a one-way ANOVA and then Tukey`s post hoc test. Values are represented by the mean ± SD (*n* = 8). NC—normal control; DEXA—dexamethasone; RGZ—rosiglitazone; GLUT—glucose transporter. A significant difference was considered at *p* < 0.05 and distinguished by * vs. NC; # vs. DEXA.

As shown in Figure 2A,B and Table S2, DEXA was associated with significantly increased cerebral glucose metabolism, as evidenced by a significant 8.3- and 9.6-fold increase in GLUT1 and GLUT3, respectively, compared with the normal control group (*p* < 0.001). In comparison to the positive control (untreated) group, there was a significant effect of RGZ (10 and 30 mg/kg for 21 days) on GLUT1, reducing its expression by 4.6- and 5.5-fold, respectively (*p* < 0.001) in the frontal brain and hippocampus. Similarly, after administration of 10 or 30 mg/kg RGZ for 21 days, GLUT3 expression was reduced by 5.5- and 6.3-fold, respectively (*p* < 0.001).

### 3.3. Reversal of the Quantity of Vital Glycolytic Enzymes in Dexamethasone-Injected Mice Brain

**Figure 3:**
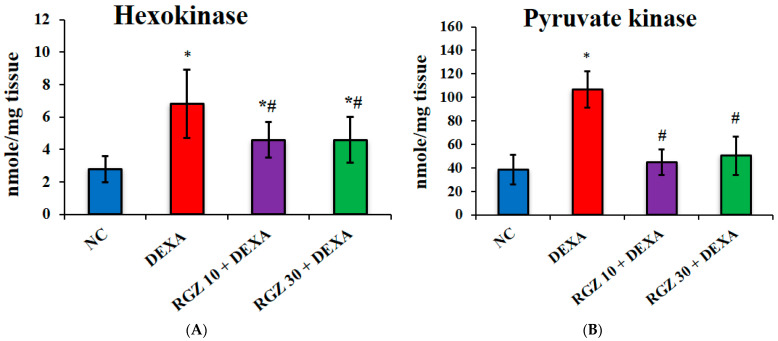
Effect of administration of DEXA, 20 mg/kg; s.c., RGZ, 10 mg/kg or 30 mg/kg, p.o., on cerebral hexokinase (**A**) and pyruvate kinase (**B**) in mice. The statistical evaluation was conducted by a one-way ANOVA and then Tukey’s post hoc test. Values are represented by the mean ± SD (*n* = 8). NC—normal control; DEXA—dexamethasone; RGZ—rosiglitazone. A significant difference was considered at *p* < 0.05 and distinguished by * vs. NC; # vs. DEXA.

The concentration of the two key glycolytic enzymes, hexokinase and pyruvate kinase, was measured in the hippocampus and frontal cortex of the experimental mice. A one-way ANOVA showed differences in the concentrations of hexokinase (*F*_3,31_ = 23.8, *p* < 0.001) and pyruvate kinase (*F*_3,31_ = 12.3, *p* < 0.001) between the different groups. In the frontal cortex and hippocampus, DEXA injection significantly increased the concentration of hexokinase and pyruvate kinase by 58% and 63%, respectively, compared with the normal control group (*p* < 0.001). Compared with the untreated group, 21-day administration of 10 mg/kg RGZ significantly lowered the concentrations of hexokinase and pyruvate kinase enzymes in the frontal cortex and hippocampus by 32% (*p* = 0.015) and 57% (*p* < 0.001), respectively. When RGZ 30 mg/kg was administered, similar results were observed, with significant decreases in hexokinase and pyruvate kinase enzymes (32%, *p* = 0.01 and 53%, *p* < 0.001, respectively). Evidently, administration of 10 and 30 mg/kg RGZ had a considerable impact and restored the pyruvate kinase levels back to their levels in the negative control group (Figure 3A,B and Table S3).

### 3.4. Reversal of mTOR Activity by Regulation of the pAMPK/pAKT/P38MAPK/4EBP1 Pathway in Dexamethazone-Injected Mice Brain

**Figure 4:**
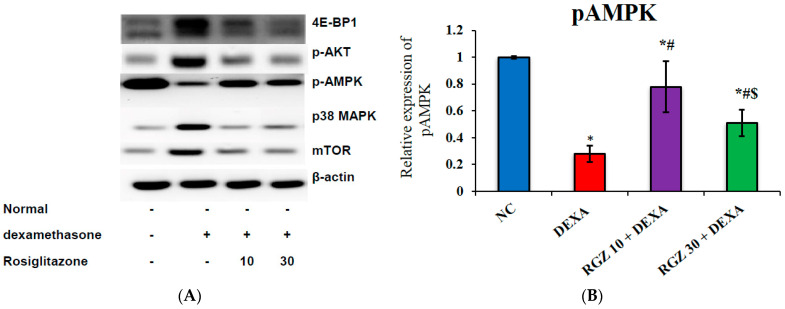

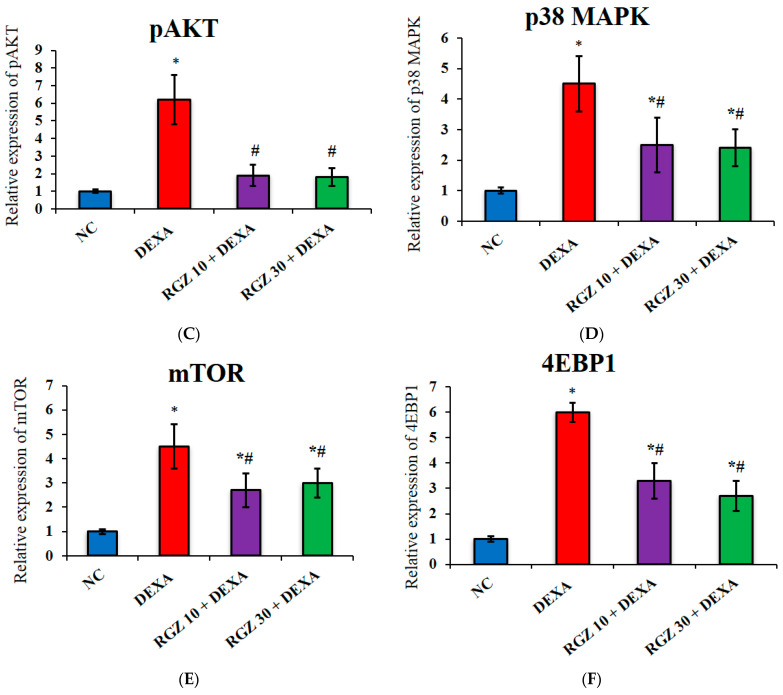
Western blot bands (**A**) representing the analysis of the Expression of (**B**) pAKT, (**C**) pAMPK-α, (**D**) p38MAPK, (**E**) mTOR, and (**F**) 4EBP1 in hippocampus and frontal cortex tissue of mice. The statistical evaluation was conducted by a one-way ANOVA and then Tukey’s post hoc test. Values are represented by the mean ± SD (*n* = 8). NC—normal control; DEXA—dexamethasone; RGZ—rosiglitazone; pAMPK—Adenosine monophosphate-activated protein kinase; pAKT—protein kinase B; p38MAPK—mitogen-activated protein kinases; mTOR—mammalian target of rapamycin complex; 14EBP1—eukaryotic initiation factor 4E (eIF4E)-binding proteins. A significant difference was considered at *p* < 0.05 and distinguished by * vs. NC; # vs. DEXA; $ vs. RGZ 10 mg/kg.

The effect of different RGZ doses on the levels of pAMPK at Thr172, an indicator of low glucose concentration, pAKT at Ser473, p38MAPK at Thr180/Tyr182, 4EBP1, and mTOR was investigated by Western blotting analysis (Figure 4A).

A significant difference in pAMPK levels was detected in the different groups (*F*_3,31_ = 70, *p* < 0.001). In comparison to the normal control group, DEXA significantly reduced the expression of pAMPK at Thr172 in the hippocampus and frontal brain by 72% (*p* < 0.001). Contrarily, administration of RGZ at a dose of 10 or 30 mg/kg for 21 days resulted in a significant increase in pAMPK at Thr172 by 64%, *p* < 0.001, and 45%, *p* = 0.001, respectively, compared with the untreated group (*p* < 0.001) (Figure 4B and Table S4).

Likewise, a significant difference was observed in the expression of pAKT at Ser473 (*F*_3,31_ = 29.7, *p* < 0.001) and p38MAPK at Thr180/Tyr182 (*F*_3,31_ = 38.6, *p* < 0.001) between the different groups. A post hoc analysis showed that DEXA significantly increased pAKT at Ser473 and p38MAPK at Thr180/Tyr182 by 83% and 77%, respectively (*p* < 0.001), as compared with the normal control group. Compared with the positive control (untreated) group, administration of 10 or 30 mg/kg RGZ significantly reduced pAKT at Ser473 by 69% and 71%, respectively (*p* < 0.001) and p38MAPK at Thr180/Tyr182 by 44% and 47%, respectively (*p* < 0.001) (Figure 4C,D and Table S4).

A one-way ANOVA analysis showed a significant difference in levels of mTOR (*F*_3,31_ = 47, *p* < 0.001) and 4EBP1 (*F*_3,31_ = 179, *p* < 0.001) in the experimental groups. In the untreated DEXA group, mTOR and 4EBP1 showed a marked increase of 77% and 83%, respectively (*p* < 0.001) compared with the normal control group. In the groups receiving 10 or 30 mg/kg RGZ, post hoc analysis showed a significant decrease in mTOR by 40% and 33%, respectively, and in 4EBP1 by 45% and 55%, respectively (*p* < 0.001) (Figure 4E,F and Table S4).

### 3.5. Reversal of Nerve Growth Factor in the Brain of Dexamethasone-Injected Mice

**Figure 5:**
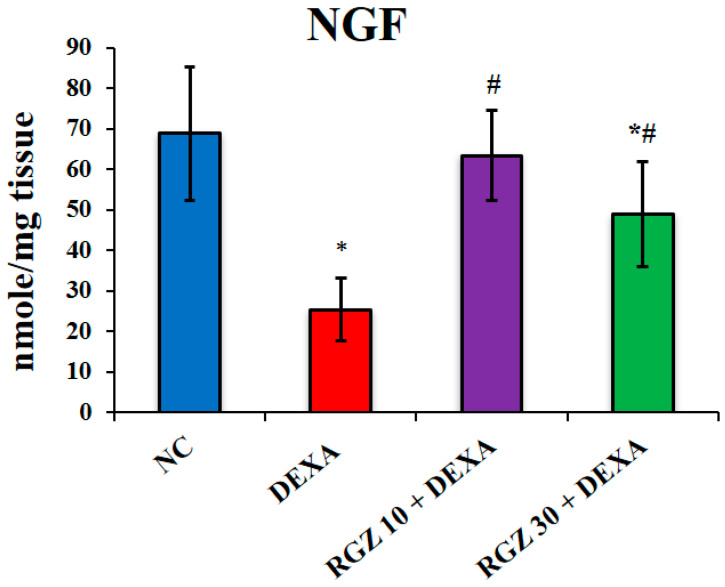
Effect of administration of DEXA, 20 mg/kg; s.c., RGZ, 10 mg/kg or 30 mg/kg p.o, on cerebral NGF in mice. The statistical evaluation was conducted by a one-way ANOVA and then Tukey`s post hoc test Values are represented by the mean ± SD (*n* = 8). NC—normal control; DEXA—dexamethasone; RGZ—rosiglitazone; NGF—nerve growth factor. A significant difference was considered at *p* < 0.05 and distinguished by * vs. NC; # vs. DEXA.

NGF is an essential factor for neuroregeneration, and NGF levels are significantly lower in major depression [53]. A one-way ANOVA analysis revealed a significant difference in NGF levels between the different groups (*F*_3,31_ = 22, *p* < 0.001). DEXA administration reduced NGF levels in the frontal cortex and hippocampal tissues by 63% versus the normal group (*p* < 0.001). After 21 days of treatment with RGZ 10 mg/kg and 30 mg/kg, these effects had been significantly reversed, as demonstrated by increases in NGF levels of 60% (*p* < 0.001) and 48% (*p* = 0.001), respectively, with nearly normal results obtained by injection of 10 mg/kg RGZ, as compared to untreated mice (Figure 5 and Table S4).

Overall, DEXA administration to mice resulted in increased immobility and depressive behavior, increased GLUT1 and GLUT3 expression, and increased hexokinase and pyruvate kinase levels in the brain. These results were associated with decreased pAMPK expression and increased mTOR, pAKT, p38MAPK, 4EBP1, and NGF expression, which may reduce neuronal autophagy. Administration of both doses of RZG (10 mg/kg or 30 mg/kg) stimulated pAMPK and simultaneously inhibited the pAKT/p38MAPK/mTOR pathway. The results suggest that 10 mg/kg RGZ has a potentiating effect on restoring normal NGF levels.

## 4. Discussion

Treatment of depression is so difficult in large part because there is no specific target that has a long-term antidepressant effect. In addition, many people with depression do not tolerate antidepressants, even at low doses. Patients often try multiple medications to control the symptoms of depression, but unacceptable side effects usually occur. Severe side effects of antidepressants, such as the regularly prescribed selective serotonin reuptake inhibitors (SSRIs), can lead to a potentially fatal condition known as “serotonin syndrome [54]. Here, we investigated the antidepressant effects of RGZ and its mechanism of action.

Depression has been linked to high glucose levels in the frontal lobe and hippocampus [19]. These higher glucose concentrations are most likely caused by increased glucose uptake due to an increased number of glucose transporters [55]. Previously, DEXA-induced depression in male mice was found to increase GLUT1 and glucose extraction in the brain compared to controls [56]. In agreement with previous studies, administration of DEXA at a dose of 20 mg/kg for 21 days was able to induce depression with an increase in the expression of GLUT1 and GLUT3 compared to normal mice. Indeed, DEXA-injected mice showed depression-like behaviors, including weight changes and immobility.

Increased cerebral glucose metabolism has been linked to depression onset, according to earlier research [57]. Neurons in the hippocampus require more energy in reaction to mental or emotional stress. In order to maintain the high ATP production in firing neurons, intracellular glucose and glycolysis are enhanced. Additionally, a prior study found that an increase in glycolytic enzymes causes an increase in glycolysis in the hippocampus and frontal brain of animal depression models [55]. In this regard, the current study demonstrated that untreated depressed rats given DEXA had higher levels of the vital glycolytic enzymes hexokinase and pyruvate kinase compared to normal mice.

Signals indicative of positive energy balance, such as high glucose concentrations and a decreased AMP/ATP ratio, inhibit AMPK [58]. Similarly, our results showed that pAMPK expression was significantly decreased in the brain tissue of mice administered DEXA compared to negative control mice. pAMPK is associated with mediators of cell cycle progression (i.e., p53), protein synthesis and growth (i.e., mTOR); elongation factor 2 kinase (eEF2K), proliferation, p38MAPK, and surviving (i.e., AKT) [59,60,61,62,63].

Animal models of depression have shown symptoms of decreased autophagy. For example, prolonged unpredictable stress decreased the levels of autophagy indicators [35,36]. Autophagy is one of the primary pathways of neurodegeneration in which mTOR and pAMPK interact, and it may have protective effects by degrading toxic, misfolded, or damaged proteins as well as damaged organelles [64]. In a previous study, it was reported that treatment of neuronal cells with Alisertib induced autophagy by increasing AMPK levels and inhibiting mTOR [65]. In response to insufficient cellular energy production, the canonical pAMPK-mTOR pathway (negatively regulated) is activated, leading to autophagy [66]. Additionally, prior research has demonstrated that a drop in glucose levels activates pAMPK, which phosphorylates Raptor, blocking mTORC1 and inducing autophagy [67,68,69]. In this context, our study showed that the induction of stress by DEXA administration was associated with decreased pAMPK protein expression and concomitantly increased mTOR expression, indicating decreased autophagy in the mice’s frontal cortex and hippocampus.

Together with mTOR activity, the AKT/TSC1-TSC2 pathway may control cell division and growth. TSC2 exhibits GTPase activity. TSC2 represses the small GTPase Rheb, which is essential for the activation of mTORC1. TSC2 is phosphorylated by AKT, resulting in the loss of TSC2′s ability to inhibit mTORC1 and activate mTOR. AKT can completely suppress TSC2 and activate mTOR by blocking pAMPK. A prior study also revealed that administration of the pAMPK activator aminoimidazolecarboxamide ribonucleotide (AICAR) led to inactivation of AKT in mice exposed to stress [70]. Moreover, the pathophysiology of depression has been related to AKT, another upstream target of mTOR [71,72]. This suggests that pAMPK activation causes autophagy activation through inhibition of mTOR [70]. Consistent with previous studies, mice stressed by DEXA injection in the current study had significantly higher pAKT protein expression than normal mice, supporting the hypothesis that depression, which is associated with many factors, activates mTOR, leading to a decrease in autophagy.

In addition, active pAMPK was found to decrease mTORC1 activity both directly and indirectly in response to energy stress and to limit amino acid consumption. Active AMPK, as previously mentioned, induces cellular autophagy, which converts excess or defective proteins into amino acids, thus replenishing the cellular amino acid pool [73]. Active AMPK can reduce the quantity of protein produced by phosphorylating and then inhibiting eukaryotic translation elongation factor 2 (eEF2) kinase, an enzyme that regulates protein synthesis. According to recent research, DEXA-stressed mice showed higher expression of the protein 4EBP1 than normal mice, suggesting an accumulation of defective proteins in the brain tissue of depressed mice.

According to previous reports, in response to inflammatory signals, p38MAPK directly and significantly aids in the restoration of autophagy regulation. In response to lipopolysaccharide stimulation in microglia, p38MAPK directly phosphorylates UNC51-like kinase-1 (ULK1), the serine/threonine kinase in the initiation complex of the autophagy cascade. Autophagy flux and level were reduced as a result of p38MAPK’s phosphorylation of ULK1, which also reduced its activity and disturbed its connection with the autophagy-related protein 13 (ATG13) in the autophagy start complex [74]. In this context, we recorded increased protein expression of p38MAPK in depressed DEXA-injected mice compared to normal mice.

Moreover, NGF-mediated autophagy is controlled by the p75NTR/AMPK/mTOR pathway in Schwann cells during Waller’s degeneration [75]. In this regard, the current study found a significantly decreased NGF concentration in the brain tissue of DEXA-injected mice compared with the normal control group, which enhanced the decreased autophagy in the brain tissue of depressed, untreated mice.

In the current research, RGZ was found to reverse depressive behavior in FST and TST. Zhao et al. [76] presented RGZ as an antidepressant that ameliorates these depressive behaviors compared with fluoxetine, an effective antidepressant, and activates autophagy by increasing the expression of LKB1 and phosphorylation of AMPK in neurons. However, the effect of RGZ on cerebral glucose metabolism, which may be involved in its antidepressant mechanism, is unknown. One of the most effective autophagy inducers, caloric restriction, has been demonstrated to be an antidepressant in rats and humans [77]. The brain needs a constant supply of glucose from the blood since it cannot store it and is so energy hungry. Whether or not hypoglycemia promotes the expression of GLUT1 and GLUT3 is uncertain and debatable [78]. In addition, a sharp decline in blood sugar levels has the potential to cause the neuroglycopenia symptoms of the “hypoglycemia phenomenon.” Although the precise process is unknown, it may be connected to the downregulation of GLUT1 and GLUT3 [79]. Consistent with previous reports, our findings indicated that the administration of 10 or 30 mg/kg RGZ for 21 days decreased cerebral glucose metabolism, as evidenced by a decrease in the expression of GLUT1 and GLUT3 and the levels of the key glycolytic enzymes hexokinase and pyruvate kinase compared with untreated mice.

A previous study in a model of global cerebral ischemia showed that RGZ had a neuroprotective function by inhibiting autophagic cell death [80]. In harmony, the present study’s findings revealed that RGZ at both doses repressed mTOR but enhanced the pAMPK protein expression in the frontal cortex and hippocampus of the mice as compared to the DEXA group, indicating increased autophagy in RGZ-treated mice. Moreover, our results showed that RGZ could affect autophagy in brain tissue by negatively regulating other upstream targets of mTOR, where RGZ reversed the DEXA-induced induction of pAKT and p38MAPK.

In a previous study, protein synthesis was found to be controlled by two known mTORC1 targets, p70 ribosomal protein S6 kinase (P70S6K) and 4EBPs, which cause elongation and translation initiation, respectively [81]. Our results showed that the low expression of mTOR achieved by RGZ administration was accompanied by downregulation of 4EBP1, suggesting a deficiency of a dysfunctional protein in brain tissue.

Recently, Li et al. [75] found that NGF suppresses mTOR activation after nerve injury, which in turn activates autophagy. In the current study, treatment of depressed mice with 10 and 30 mg/kg RGZ increased NGF levels in hippocampal and frontal cortex tissues compared with untreated mice.

The present study does not fully explain the detailed mechanism of RGZ in the animal model of depression. Nevertheless, our findings show that RGZ is a prospective therapeutic approach for MDD, the pathogenesis of which is associated with increased glucose metabolism.

In addition, forthcoming studies are recommended to highlight the effect of RGZ compared with approved pharmacological treatments and to explore safe and effective drugs for the treatment of MDD, such as RGZ.

## 5. Conclusions

Overall, this study concluded that RGZ significantly improved depressive behavior in depressed mice. Based on the positive relationship between depression and glucose metabolism, DEXA administration was found to induce MDD-like pathology through high glucose metabolism associated with increased expression of GLUT1 and GLUT3, along with increased levels of hexokinase and pyruvate kinase in the brain. These findings have been associated with decreased expression of pAMPK and upregulation of mTOR, pAKT, p38MAPK, 4EBP1, and NGF, which in turn may reduce neuronal autophagy. Indeed, the current study provides evidence that RGZ can enhance and maintain protective autophagy in the brain by decreasing the AMP/ATP ratio, activating pAMPK, and inhibiting the pAKT/p38MAPK/mTOR pathway. Our study duly considered the importance of the role of NGF in the neuroprotective effect of RGZ, considering the potentiating effect of 10 mg/kg RGZ in restoring normal NGF levels.

## Figures and Tables

**Table 1:** Primer sequences of the assessed genes used in RT-qPCR.

Gene ID	Primers Sequence (5′→3′)		Annealing Temperature
	Forward	Reverse	
GLUT1	CCAGCTGGGAATCGTCGTT	CAAGTCTGCATTGCCCATGAT	62 °C
GLUT3	CTCTTCAGGTCACCCAACTACGT	CCGCGTCCTTGAAGATTCC	55 °C
β-actin	ACGGCCAGGTCATCACTATTG	CAAGAAGGAAGGCTGGAAAAGA	56 °C

GLUT: glucose transporter.

## Data Availability

Data is available within the article and Supplementary Materials.

## Supplementary Materials

The following supporting information can be downloaded at: https://www.mdpi.com/article/10.3390/biomedicines11030860/s1. Table S1. Effect of rosiglitazone on depression behavior; Table S2. Effect of rosiglitazone on brain glucose transporters-1 and 3; Table S3. Effect of rosiglitazone on brain glycolytic enzymes; Table S4. Effect of rosiglitazone on mTOR/pAMPK/pAKT/P38MAPK/4EBP1 pathway and nerve growth factor.
