# Supplementary material for: Rosiglitazone Mitigates Dexamethasone-Induced Depression in Mice via Modulating Brain Glucose Metabolism and AMPK/mTOR Signaling Pathway
Source: Biomedicines. 2023 Mar 11;11(3):860. doi: 10.3390/biomedicines11030860 (PMC10046017; doi:10.3390/biomedicines11030860)
Supplement: Supplementary file 1 [file biomedicines-11-00860-s001.zip › biomedicines-2195474-supplementary.pdf]

Table S1. Effect of rosiglitazone on depressive behavior.

**Groups**	**FST** Immobility time (seconds)	***P*-value**	**TST** Immobility time (seconds)	***P*-value**
NC	119 ± 11.3		144 ± 13.1	
DEXA	168 ± 23.3	< 0.001*	214 ± 37.5	< 0.001*
DEXA + (RGZ 10 mg/kg)	140 ± 15	0.034*<br>0.003#	174.7 ± 31.3	0.012#
DEXA + (RGZ 30 mg/kg)	138.2 ± 13.2	0.001#	154.8 ± 16.8	< 0.001#

Comparisons between different groups were performed by a one-way ANOVA test, followed by Tukey's post hoc test for multiple comparisons. Data are presented as mean ± SD. NC—normal control; DEXA—dexamethasone; RGZ—rosiglitazone; FST—forced swimming test; TST—tail suspension test. * indicates significant difference vs. NC, # indicates significant difference vs. DEXA.

Table S2. Effect of rosiglitazone on brain glucose transporters-1 and 3.

**Groups**	**GLUT1** Fold change	***P*-value**	**GLUT3** Fold change	***P*-value**
NC	1 ± 0.1		1 ± 0.1	
DEXA	8.3 ± 1.9	< 0.001*	9.6 ± 2.5	< 0.001*
DEXA + (RGZ 10 mg/kg)	4.6 ± 1.2	< 0.001*<br>< 0.001#	5.5 ± 1.5	< 0.001*<br>< 0.001#
DEXA + (RGZ 30 mg/kg)	5.5 ± 0.9	< 0.001*<br>< 0.001#	6.3 ± 1.1	< 0.001*<br>< 0.001#

Comparisons between different groups were performed by a one-way ANOVA test, followed by Tukey's post hoc test for multiple comparisons. Data are presented as mean ± SD. NC—normal control; DEXA—dexamethasone; RGZ—rosiglitazone; GLUT—glucose transporter. * indicates significant difference vs. NC, # indicates significant difference vs. DEXA.

Table S3. Effect of rosiglitazone on brain glycolytic enzymes.

**Groups**	**Hexokinase** nmole/mg	***P*-value**	**Pyruvate kinase** nmole/mg	***P*-value**
NC	2.8 ± 0.8		38.4 ± 12.5	
DEXA	6.8 ± 2.1	< 0.001*	106.5 ± 15.5	< 0.001*
DEXA + (RGZ 10 mg/kg)	4.6 ± 1.1	0.034*<br>0.015#	45 ± 11	< 0.001#
DEXA + (RGZ 30 mg/kg)	4.6 ± 1.4	0.04*<br>0.01#	50.3 ± 16.3	< 0.001#

Comparisons between different groups were performed by a one-way ANOVA test, followed by Tukey's post hoc test for multiple comparisons. Data are presented as mean ± SD. NC—normal control; DEXA—dexamethasone; RGZ—rosiglitazone. * indicates significant difference vs. NC, # indicates significant difference vs. DEXA.

Table S4. Effect of rosiglitazone on the mTOR/pAMPK/pAKT/P38MAPK/4EBP1 pathway and nerve growth factor.

Groups	NC	DEXA	DEXA + (RGZ 10 mg/kg)	DEXA + (RGZ 30 mg/kg)
**pAMPK** (relative intensity)	1 ± 0.01	0.28 ± 0.06<br>*P < 0.001**	0.78 ± 0.19<br>*P = 0.001**<br>*P < 0.001#*	0.51 ± 0.1<br>*P < 0.001*, 0.001#,*<br>*P < 0.001$*
**pAKT** (relative intensity)	1 ± 0.1	6.2 ± 1.4<br>*P < 0.001**	1.9 ± 0.6<br>*P < 0.001#*	1.8 ± 0.5<br>*P < 0.001#*
**p38MAPK** (relative intensity)	1 ± 0.1	4.5 ± 0.9<br>*P < 0.001**	2.5 ± 0.9<br>*P < 0.001*,*<br>*P < 0.001#*	2.4 ± 0.6<br>*P= 0.001*,*<br>*P < 0.001#*
**4EBP1** (relative intensity)	1 ± 0.1	6 ± 0.37<br>*P < 0.001**	3.3 ± 0.7<br>*P < 0.001*,*<br>*P < 0.001#*	2.7 ± 0.6<br>*P < 0.001*,*<br>*P < 0.001#*
**mTOR** (relative intensity)	1 ± 0.1	4.5 ± 0.9<br>*P < 0.001**	2.7 ± 0.7<br>*P < 0.001*,*<br>*P < 0.001#*	3 ± 0.6<br>*P < 0.001*,*<br>*P < 0.001#*
**NGF** (nmole/mg)	68.8 ± 16.4	25.4 ± 7.7<br>*P < 0.001**	63.4 ± 11.1<br>*P < 0.001#*	49 ± 12.9<br>*P = 0.02**<br>*P = 0.001#*

Comparisons between different groups were performed by a one-way ANOVA test, followed by Tukey's post hoc test for multiple comparisons. Data are presented as mean ± SD. NC—normal control; DEXA—dexamethasone; RGZ—rosiglitazone; pAMPK—Adenosine monophosphate-activated protein kinase; pAKT—protein kinase B; p38MAPK—mitogenactivated protein kinases; mTOR—mammalian target of rapamycin complex; 4EBP1—eukaryotic initiation factor 4E (eIF4E)-binding proteins; NGF—nerve growth factor. * indicates significant difference vs. NC, # indicates significant difference vs. DEXA, $ indicates significant difference vs. RGZ (10 mg/kg).
